# Sex- and cycle-dependent changes in spine density and size in hippocampal CA2 neurons

**DOI:** 10.1038/s41598-024-62951-x

**Published:** 2024-05-28

**Authors:** Sharif Jabra, Michael Rietsche, Julia Muellerleile, Aet O’Leary, David A. Slattery, Thomas Deller, Meike Fellenz

**Affiliations:** 1 Institute of Clinical Neuroanatomy, Neuroscience Center, Goethe University Frankfurt, Theodor-Stern-Kai 7, 60590 Frankfurt am Main, Germany; 2 Department of Psychiatry, Psychosomatic Medicine and Psychotherapy, Goethe University Frankfurt, University Hospital, Heinrich-Hoffmann-Straße 10, 60528 Frankfurt am Main, Germany

**Keywords:** Spine, Estrogen, Estrous cycle, Structural plasticity, CA2, CA1, CA3, Hippocampus, Cellular neuroscience, Synaptic plasticity

## Abstract

Sex hormones affect structural and functional plasticity in the rodent hippocampus. However, hormone levels not only differ between males and females, but also fluctuate across the female estrous cycle. While sex- and cycle-dependent differences in dendritic spine density and morphology have been found in the rodent CA1 region, but not in the CA3 or the dentate gyrus, comparable structural data on CA2, i.e. the hippocampal region involved in social recognition memory, is so far lacking. In this study, we, therefore, used wildtype male and female mice in diestrus or proestrus to analyze spines on dendritic segments from identified CA2 neurons. In basal *stratum oriens*, we found no differences in spine density, but a significant shift towards larger spine head areas in male mice compared to females. Conversely, in apical *stratum radiatum* diestrus females had a significantly higher spine density, and females in either cycle stage had a significant shift towards larger spine head areas as compared to males, with diestrus females showing the larger shift. Our results provide further evidence for the sexual dimorphism of hippocampal area CA2, and underscore the importance of considering not only the sex, but also the stage of the estrous cycle when interpreting morphological data.

## Introduction

For decades, the small hippocampal area CA2 was neglected as an unimportant transition zone between the CA3 and CA1. However, recent studies examining marker proteins, electrophysiological properties and connectivity of CA2 pyramidal cells have redefined the borders of CA2^1–3^, and it is now recognized as a larger, discrete hippocampal subregion with specialized functions. CA2 is crucially involved in social recognition memory^4^, the ability to remember a conspecific. Interestingly, while both male and female rodents display a preference for unfamiliar conspecifics, male rats^5^ and mice^6^ spent more time investigating an unfamiliar conspecific than females, whereas female rats demonstrated longer-lasting social memory^5^. In addition, CA2 has recently been implicated in contextual fear conditioning. Female, but not male, mice responded with increased freezing behavior to experimentally-induced changes of CA2 activity^7^, once more highlighting a sexual dimorphism of CA2 function.

Sex hormones are known to influence hippocampal synaptic plasticity and dendritic spine morphology in rodents^8–12^. In sexually mature females, the plasma levels of estradiol, progesterone, and luteinizing hormone change constantly across the estrous cycle, and Woolley et al. were the first to show that apical dendritic spine density in *stratum lacunosum-moleculare* of rat CA1 pyramidal neurons fluctuates significantly across the stages of estrous^13^. These results have been confirmed by other groups^14,15^, and more recent studies also found evidence for cyclic changes in spine density in apical CA1 *stratum radiatum*^16^ and in distal-most branches of basal dendrites sampled from CA1 and CA2 *stratum oriens*^17^ in rats. Interestingly, no cyclicity of spine density was found in CA3 pyramidal neurons or dentate gyrus granule cells of rats^13^, or in cortical neurons of mice^18,19^. While significant cycle-dependent changes in overall spine density have not been reported to date in the hippocampus of wildtype mice, Brandt et al. found that it was specifically the density of large spines on apical CA1 pyramidal cell dendrites that significantly fluctuated across the estrous cycle of female mice^20^. Thus, several factors including the species, the hippocampal subregion, and even the neuronal layers appear to determine the estrous cyclicity of spine density and size^21^. As spines represent the postsynaptic site of the majority of excitatory neurotransmission in the brain^22^, a change in spine density presumably impacts the amount of excitatory neurotransmission, and thus might change information processing in a given region^23^. Spine morphology, in turn, has long been used as a structural readout of synaptic strength. Large spines are more resistant to structural potentiation^24^, suggesting that they represent the fraction of stable spines that make up a memory trace^25^. Therefore, sex- and/or cycle-dependent changes in the density or size of spines in specific layers or subregions could be related to memory performance in specific tasks.

Although CA2 is now well-recognized for its function in social memory^4,26^, a behavior known to be influenced by sex in rodents^5,6^, no study has so far addressed the physiological influence of sex hormones on basic spine parameters in CA2 neurons. To fill this gap, we used wildtype C57BL/6 male and female mice in either the diestrus or proestrus stage of the estrous cycle, and randomly filled single neurons in the putative CA2 region with a fluorescent dye. Subsequent immunohistochemical labeling with the CA2 marker Purkinje cell protein 4 (PCP4)^1^ enabled us to post-hoc identify single neurons as CA2. Finally, we imaged basal and apical dendritic segments in *stratum oriens* and *stratum radiatum*, respectively, and analyzed spine density and spine head size.

## Results

### Sex-dependent changes in spine head size in basal dendrites in *stratum oriens*

**Figure 1:**
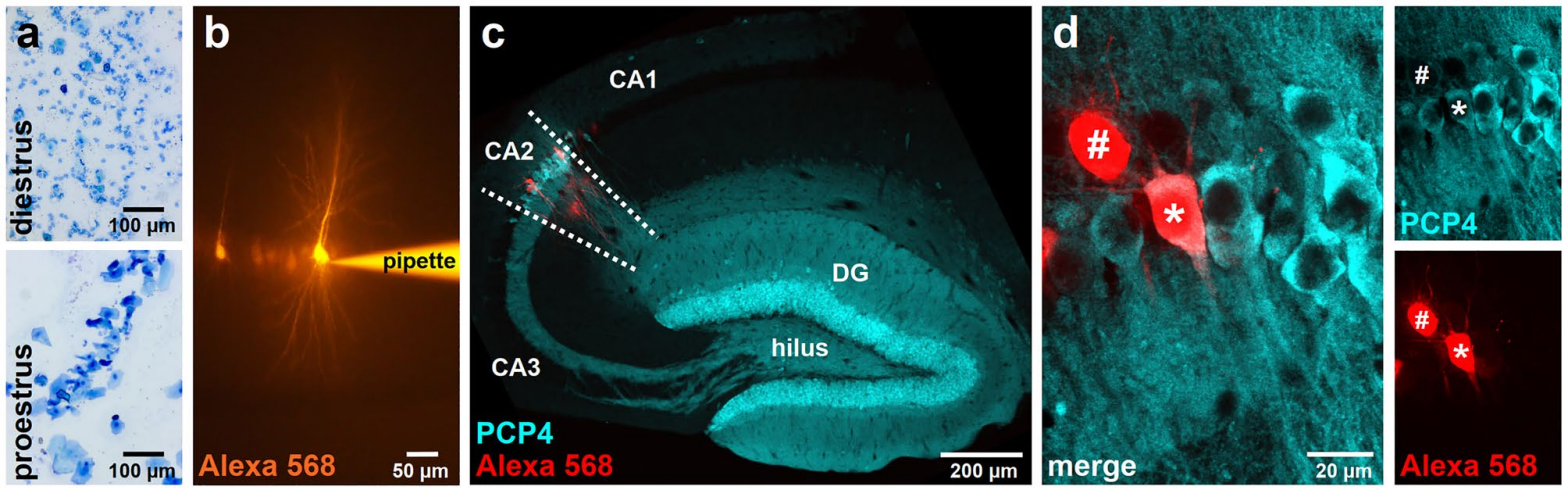
Identification of PCP4-positive CA2 neurons in the hippocampus of male mice and female mice in the diestrus and proestrus phase of the ovarian cycle. (**a**) Vaginal cytology was used to determine the phase of the cycle of experimental animals. Diestrus and proestrus were determined using the following characterization: diestrus—epithelial cells and leucocytes observed; proestrus—large nucleated epithelial cells and no leucocytes observed. (**b**) Neuron filled iontophoretically with Alexa 568 (orange). (**c**) Hippocampal section stained for PCP4 (cyan) after filling of neurons with Alexa 568 (red). PCP4 labels pyramidal cells in the CA2 region of the hippocampus as well as granule cells in the dentate gyrus (DG). (**d**) Post-hoc identification of Alexa 568-filled cells. Double-labeling with PCP4 (cyan) identifies Alexa 568-filled cells (red) as CA2 neurons (asterisk). Single channels shown on the right demonstrate the specificity of the labeling. Filled cells with PCP4-negative cell body (#) were not considered for analysis.

To investigate the effects of sex and estrous cycle stage on spine parameters in hippocampal area CA2, we used perfusion-fixed brain sections of male and female mice in either the diestrus or proestrus stage of the cycle (Fig. 1a). Compared to CA1 and CA3, CA2 is a very small region, and, therefore, harder to target specifically. In addition, the boundaries of the molecular CA2 are not clear-cut, so it is expected that non-CA2-cells are intermingled with CA2 cells at the proximal and distal borders of CA2. We, therefore, first randomly filled pyramidal neurons along the CA3-CA2-CA1 axis with the fluorescent dye Alexa 568, and then post-hoc stained for the established CA2 marker PCP4 (Fig. 1b, c) to identify CA2 neurons. While filled cells with PCP4-negative cell bodies were frequently found adjacent to filled cells with PCP4-positive cell bodies (Fig. 1d), only dendritic segments from neurons with a PCP4-positive cell body were imaged and analyzed.

**Figure 2:**
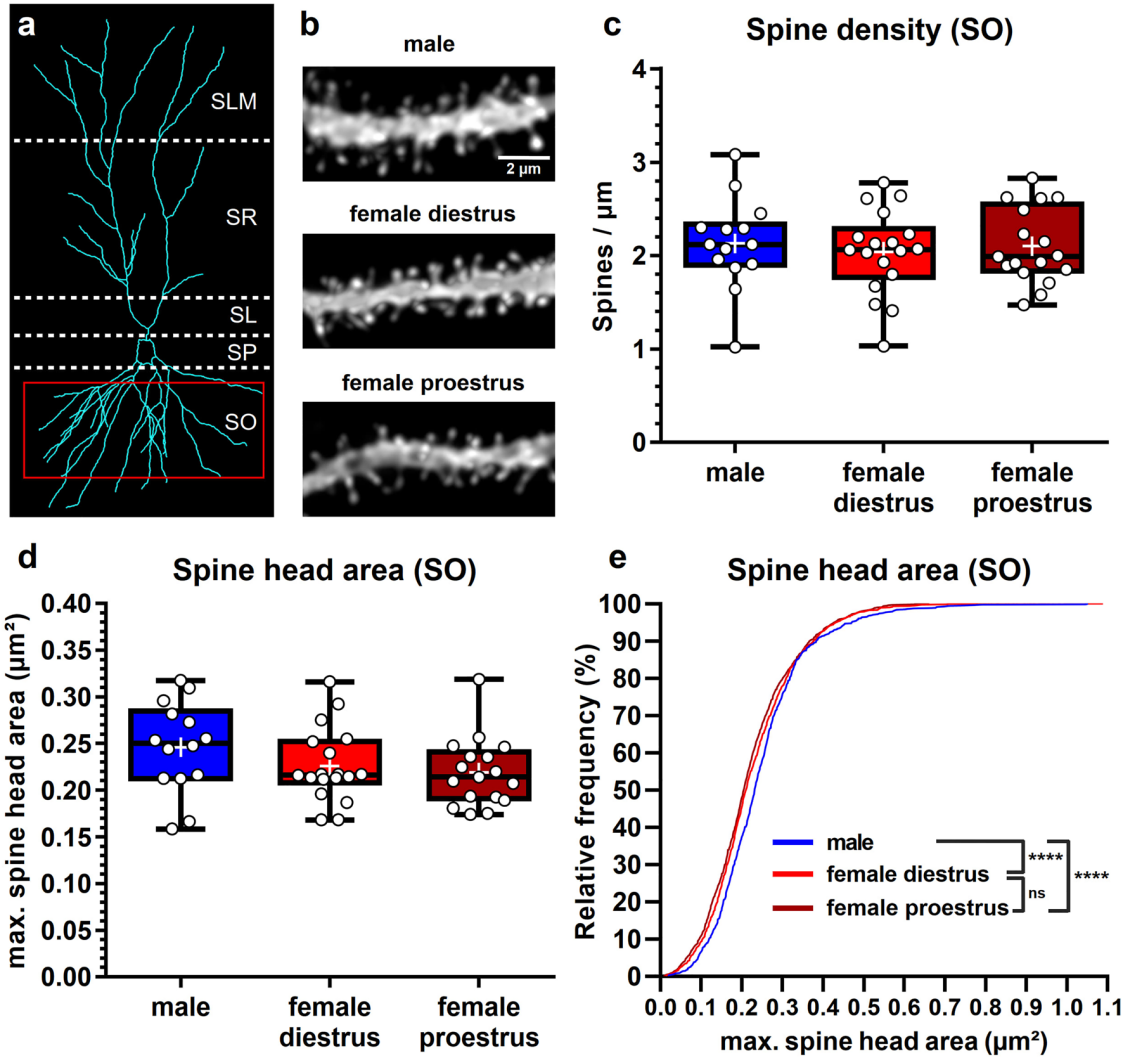
Sex-dependent differences in spine head size in *stratum oriens* of CA2. (**a**) Representative reconstruction of an Alexa Fluor 568-filled CA2 cell. Red box shows the area of *stratum oriens* (SO) in which basal dendritic segments of CA2 cells were imaged. *SP* stratum pyramidale, *SL* stratum lucidum, *SR* stratum radiatum, *SLM* stratum lacunosum-moleculare. (**b**) Representative images of deconvolved basal dendritic segments in SO of male, female diestrus and female proestrus mice. (**c**) Comparison of spine densities of basal dendritic segments in SO. Spine densities are not significantly different between male mice (2.13 ± 0.49 spines/μm), female mice in diestrus (2.04 ± 0.45 spines/µm) and female mice in proestrus (2.10 ± 0.40 spines/µm; mean ± s.d.). Kruskal–Wallis test: H(2) = 0.3443, *P* = 0.8419, ns). (d) Comparison of average spine head areas per segment in SO. Average values per segment are not significantly different between male mice (0.25 ± 0.05 μm^2^), female mice in diestrus (0.23 ± 0.04 μm^2^) and female mice in proestrus (0.22 ± 0.04 μm^2^; mean ± s.d.). Kruskal–Wallis test: H(2) = 3.066, *P* = 0.2158, ns. (**c**,**d**) n_*male*_ = 14 segments from six mice, n_*female diestrus*_ = 18 segments from seven mice, n_*female proestrus*_ = 17 segments from eight mice. (**e**) Cumulative frequency plots of spine head areas from all segments. Male mice show a highly significant shift towards larger spine head areas compared to both, females in diestrus and females in proestrus (Kolmogorov–Smirnov test; male vs female diestrus + male vs female proestrus: ****P < 0.0001 respectively. Female diestrus vs female proestrus: *P* = 0.2346, ns). n_*male*_ = 883 spine heads; n_*female diestrus*_ = 1297 spine heads; n_*female proestrus*_ = 1182 spine heads.

We first evaluated basal segments in *stratum oriens* of identified CA2 pyramidal neurons (Fig. 2a, b). Analysis of average spine density showed no significant differences between the sexes or the two different cycle stages (Fig. 2c; male: 2.13 ± 0.49 spines/μm; female diestrus: 2.04 ± 0.45 spines/µm; female proestrus: 2.10 ± 0.40 spines/µm; Kruskal–Wallis test: H(2) = 0.3443, *P* = 0.8419; ns). We then proceeded to measure the maximum cross-sectional head areas of all counted spines that displayed a head. Indeed, we were able to detect sex-dependent differences in the maximum spine head area of those spines. While these differences were not apparent when comparing only the averaged values per segment (Fig. 2d; male: 0.25 ± 0.05 μm^2^; female diestrus: 0.23 ± 0.04 μm^2^; female proestrus: 0.22 ± 0.04 μm^2^; Kruskal–Wallis test: H(2) = 3.066, *P* = 0.2158; ns), a highly significant shift towards larger spine head areas in males compared to females in either estrous cycle stage was clearly visible when looking at the cumulative frequency distribution of the area of all measured spine heads (Fig. 2e; Kolmogorov–Smirnov test; male vs female diestrus + male vs female proestrus: ****P < 0.0001; respectively; female diestrus vs female proestrus: *P* = 0.2346; ns). Collectively, our data showed no effect of sex or estrous cycle on spine densities in basal segments of CA2 neurons, but a clear effect of sex on spine head area that was independent of the stage of estrous (cf. Fig. 4).

### Sex- and cycle-dependent changes in spine density and spine head size in apical dendrites in *stratum radiatum*

**Figure 3:**
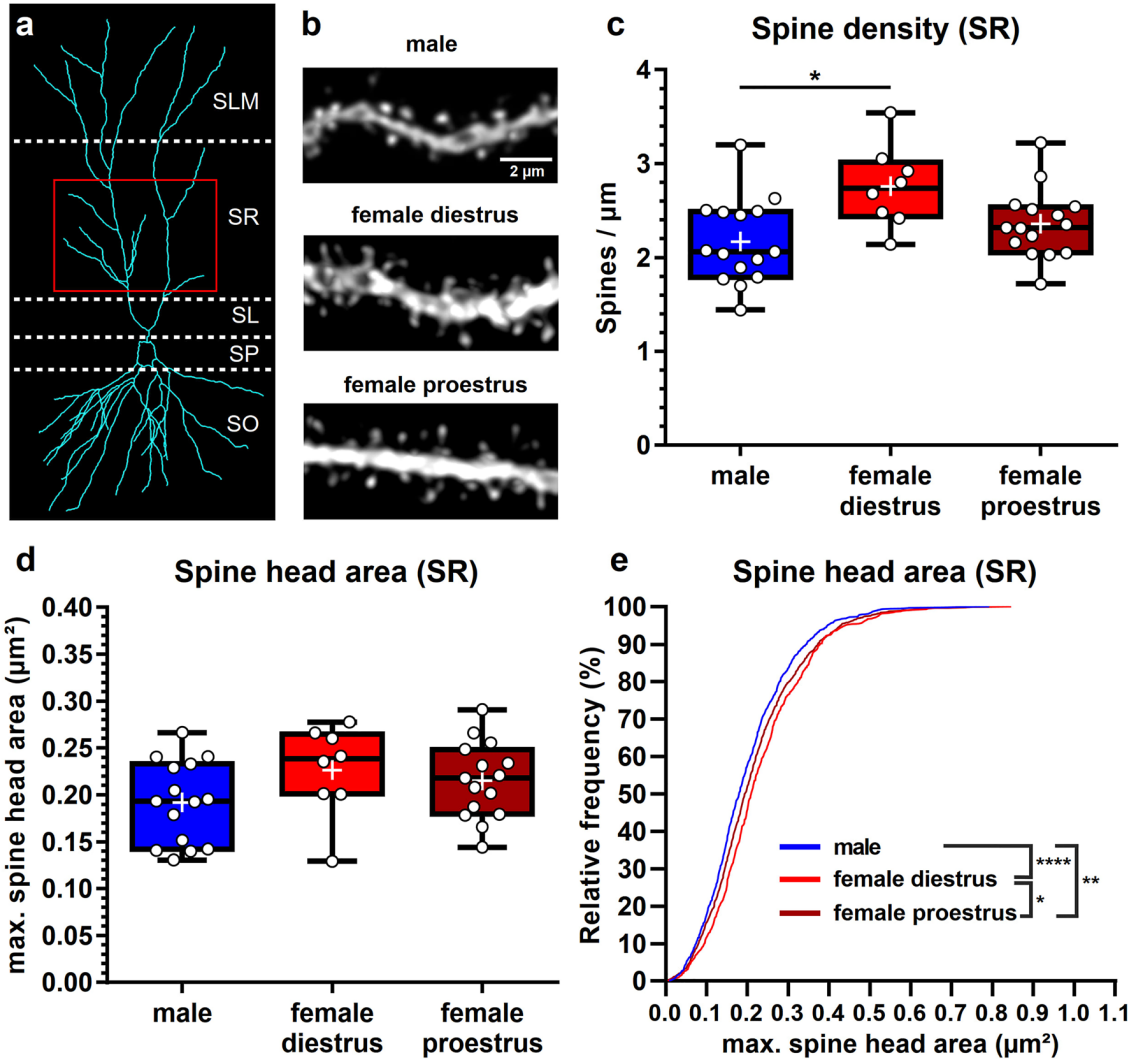
Sex- and cycle-dependent differences in dendritic spine densities and spine head sizes in *stratum radiatum* of CA2. (**a**) Representative reconstruction of an Alexa 568-filled CA2 cell. Red box shows the area of *stratum radiatum* (SR) in which apical dendritic segments of CA2 cells were imaged. SO *stratum oriens*, SP *stratum pyramidale*, SL *stratum lucidum*, SLM *stratum lacunosum-moleculare.* (**b**) Representative images of deconvolved apical dendritic segments in SR of male, female diestrus and female proestrus mice. (**c**) Comparison of spine densities of dendritic segments in SR of male mice (2.17 ± 0.45 spines/μm), female mice in diestrus (2.75 ± 0.43 spines/µm) and female mice in proestrus (2.36 ± 0.37 spines/µm; mean ± s.d.). A Kruskal-Wallis test detected differences between the three groups (Kruskal-Wallis test: H(2) = 7.898, *P* = 0.0193). Post-hoc Dunn’s multiple comparison test revealed a significant difference between male mice and female mice in diestrus (*P = 0.0148; male vs. female proestrus:* P* = 0.7401, ns; female diestrus vs. female proestrus: *P* = 0.1955, ns). (**d**) Comparison of average spine head areas per segment in SR. There is no significant difference in average spine head area between male mice (0.19 ± 0.04 μm^2^), female mice in diestrus (0.23 ± 0.05 μm^2^) and female mice in proestrus (0.22 ± 0.04 μm^2^; mean ± s.d.). Kruskal–Wallis test: H(2) = 3.677, *P* = 0.1590; ns. (**c**,**d**) n_*male*_ = 15 segments from five mice, n_*female diestrus*_ = 8 segments from four mice, n_*female proestrus*_ = 15 segments from five mice. (**e**) Cumulative frequency plots of spine head areas from all segments. Both, females in proestrus and females in diestrus, show a significant shift towards larger spine head areas as compared to males, with female mice in diestrus displaying a significantly more pronounced right-shift (Kolmogorov–Smirnov (K.–S.) test; male vs female diestrus: ****P < 0.0001; male vs female proestrus: **P = 0.0065; female diestrus vs female proestrus: *P = 0.0113). n_*male*_ = 1156 spine heads; n_*female diestrus*_ = 667 spine heads; n_*female proestrus*_ = 1534 spine heads.

**Figure 4:**
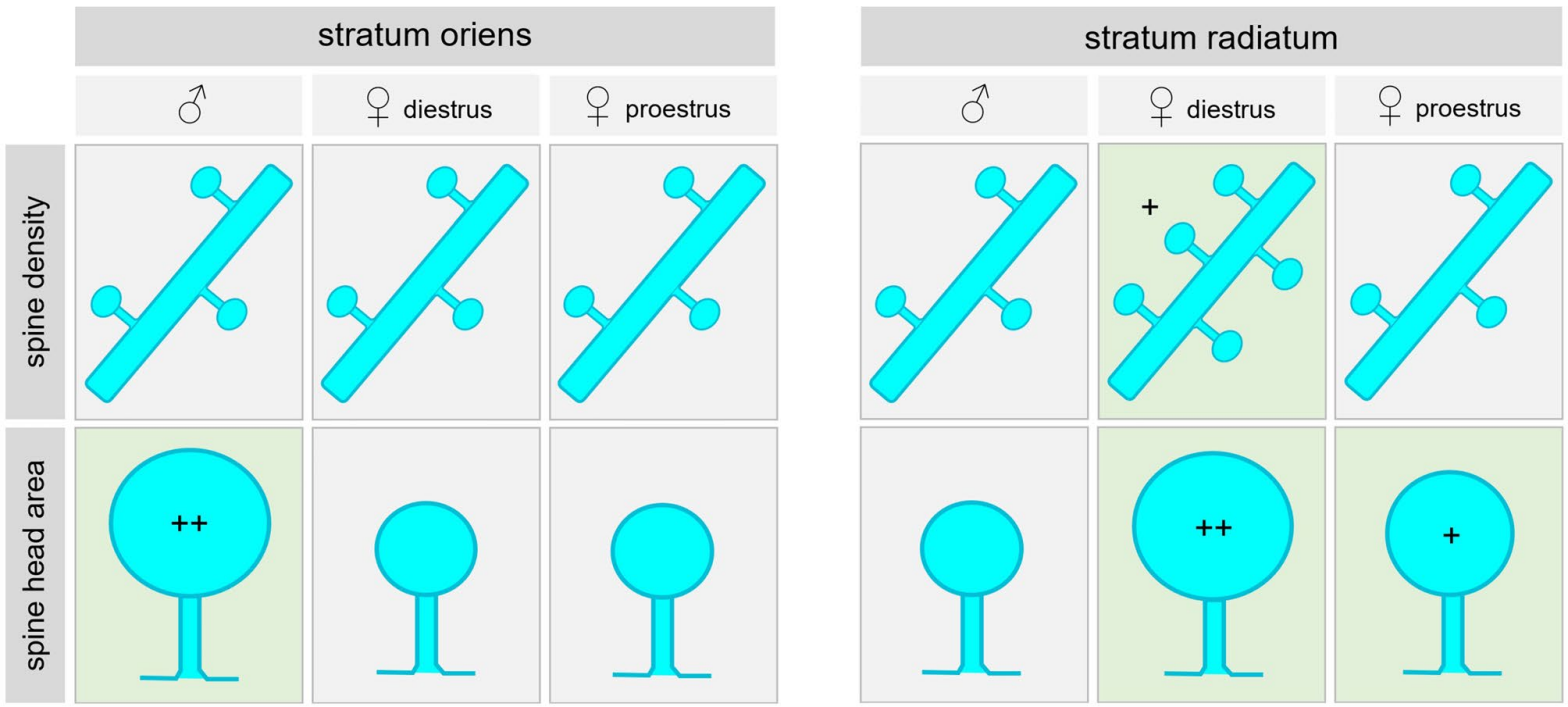
Summary of sex/cycle-dependent differences in spines of CA2 pyramidal neurons. In *stratum oriens* of CA2, sex-dependent differences in pyramidal cell spines were observed: male mice exhibited larger spine heads on basal PCP4-positive dendrites, whereas spine densities were comparable between sexes. No cycle-dependent changes were observed. In *stratum radiatum* of CA2, sex- and cycle-dependent differences in pyramidal cell spines were found: female mice in the diestrus stage of the cycle displayed a higher spine density compared to male mice, and female mice in either stage of the cycle had a higher proportion of spines with larger heads than males. In addition, spine head size distributions also showed additional cycle-dependent differences, as females in diestrus had an even higher proportion of larger spines than females in proestrus.

While the basal compartment of CA2 cells receives mainly extrahippocampal input, afferents onto apical dendrites are mostly intrahippocampal, and both compartments are also known to differ in their reaction to excitatory stimulation^27^. Accordingly, it is reasonable to assume that sex hormones can have differential effects on basal and apical dendritic segments. We, therefore, in a next step extended our analysis to apical segments in *stratum radiatum* of CA2 (Fig. 3a, b). As before, we first looked at sex- and/or cycle dependent changes in spine density. In contrast to basal segments, spine density on apical segments in *stratum radiatum* differed significantly between the three groups (Fig. 3c; male: 2.17 ± 0.45 spines/μm; female diestrus: 2.75 ± 0.43 spines/µm; female proestrus: 2.36 ± 0.37 spines/µm; Kruskal–Wallis test: H(2) = 7.898, *P* = 0.0193). Interestingly, this difference was sex-dependent, but cycle-specific, as only female animals in diestrus showed a significantly higher average spine density compared to male mice, while female animals in proestrus did not (post-hoc Dunn’s multiple comparison test: male vs. female diestrus: *P = 0.0148; male vs. female proestrus: *P* = 0.7401, ns; female diestrus vs. female proestrus: *P* = 0.1955, ns). We then analyzed the spine head areas of all spines with a discernable head. As for basal segments, only looking at the average spine head area per segment failed to unveil significant changes (Fig. 3d; male: 0.19 ± 0.04 μm^2^; female diestrus: 0.23 ± 0.05 μm^2^; female proestrus: 0.22 ± 0.04 μm^2^; Kruskal–Wallis test: H(2) = 3.677, *P* = 0.1590, ns), whereas the more sensitive cumulative frequency distribution revealed that females in diestrus as well as females in proestrus have significantly more spines with bigger heads than males. This effect was more pronounced in diestrus females, and their distribution was right-shifted significantly even when compared to proestrus females, revealing an additional influence of cycle stage on spine head area. (Fig. 3e; Kolmogorov–Smirnov test; male vs female diestrus: ****P < 0.0001; male vs female proestrus: **P = 0.0065; female diestrus vs female proestrus: *P = 0.0113). Taken together, our data showed a clear effect of sex on both spine density and spine head area. In addition, there was a cycle-dependent component to these effects, as significantly higher spine densities as compared to males were only apparent in females in diestrus, and spine head size distributions of females were not only significantly different from males, but also varied significantly between diestrus and proestrus (cf. Fig. 4).

## Discussion

We show here that dendritic spine densities and spine head sizes in CA2 pyramidal cells exhibit layer-specific sex- and cycle-dependent differences in their distributions. While spine head sizes were significantly larger in *stratum oriens* of male mice, both the spine densities and the spine head sizes in *stratum radiatum* were greater in female mice in the diestrus stage of the estrous cycle compared to females in proestrus and males.

In CA1 neurons, spine densities on apical dendrites in *stratum lacunosum-moleculare* and *stratum radiatum* have been shown to fluctuate with the estrous cycle in rats. Densities were significantly lower during estrus as compared to diestrus and proestrus, while the highest absolute values were found during proestrus^13–16^. Significant sex-dependent differences have only been shown between male rats and females in proestrus, with males having lower spine densities than proestrus females^19^. The observed differences in spine density have been attributed to the fluctuating levels of estrogen, as highest and lowest spine values coincided with phases of high and low estrogen levels during the cycle. In addition, estrogen treatment alone could significantly increase spine density in ovariectomized rats^28^. Unfortunately, spine morphology parameters have been analyzed in very few studies. To our knowledge, only Gonzales-Burgos et al.^14^ compared spine morphologies in rats, and found more mushroom-type spines in CA1 *stratum radiatum* of proestrus compared to estrus rats. Furthermore, even fewer studies have been published on the effect of the estrous cycle on spine morphology in mice. A recent study analyzing apical obliques in CA1 *stratum radiatum* in mice found no effect of sex on average spine density, but could show that it is specifically the density of large spines (head diameter > 0.6 µm) that is significantly increased in female vs. male mice^20^. Further discrimination between estrous cycle stages revealed that the density of large spines in females was highest in diestrus, not proestrus as in rats. Although the mice analyzed in that study were considerably older (8 months) than our animals (2.5–3.5 months), and potential age-related changes in spine density have to be considered, our data from CA2 *stratum radiatum* point in a similar direction. As compared to males, we also found a significant shift towards larger spine head areas in females in both proestrus and diestrus, with the larger increase found in diestrus. The results obtained from rats and mice might seem contradictory at first. Yet, while it is widely agreed on that estrogen levels peak in proestrus, fall during estrus, and then gradually rise again during the diestrus stages in rats^29–32^, reported estrogen profiles across the estrous cycle have been inconsistent in different strains of mice^33–36^. Interestingly, however, a very recent study investigated plasma levels of gonadal hormones in C57BL/6J mice—which were also used in this study—on a thrice-a-day basis, and found an unexpected and hitherto unreported estrogen surge at 10 a.m. during diestrus^37^. This might explain why spine densities seem to be highest in diestrus, not proestrus, in the mouse studies.

CA1 and CA2 neurons receive the same afferents in *stratum radiatum*, namely, the Schaffer collaterals from CA3 pyramidal cells. However, while CA3-CA1 synapses exhibit robust long-term potentiation (LTP), CA3-CA2 synapses are highly resistant to LTP^38,39^. It has been proposed that perineuronal nets (PNNs), a specialized form of extracellular matrix surrounding *stratum radiatum* dendrites and synapses of pyramidal cells in CA2, but not CA1, might account for this difference in plasticity^38^. A recent study, also conducted in C57BL/6 mice, provided evidence for a cycle-dependent fluctuation in the number of PNN-enwrapped parvalbumin-positive interneurons in the ventral dentate gyrus, with the lowest numbers occurring during diestrus^40^. This raises the possibility that cyclic changes in sex hormones might regulate PNNs, and, thereby, also influence synaptic plasticity and spine size in CA2 *stratum radiatum*. The molecular mechanisms underlying the estrous cyclicity of the PNNs are not yet clear, though there is evidence for the modulation of PNNs by sex hormones. Laham et al. showed that inhibiting the progesterone metabolite allopregnanolone during diestrus significantly increased the number of PNN-enwrapped cells in ventral CA1^40^, while Hernandez–Vivanco et al. observed an increase in the intensity of PNNs surrounding parvalbumin-positive interneurons in dorsal CA1 of female (non-cycle staged) mice after blocking aromatase activity^41^.

The changes we observed in spine density and shape were remarkably layer-specific, as in *stratum oriens* it was the male mice, not the females, that had a shifted distribution towards larger spine head areas, but there was no apparent effect of the stages of estrous investigated. Indeed, CA2 pyramidal cells receive a variety of intra- and extrahippocampal afferents that innervate distinct layers. While, as discussed above, both CA2 and CA1 cells are predominantly innervated by CA3 Schaffer collaterals in *stratum radiatum*, innervation of basal *stratum oriens* in CA2 is mainly extrahippocampal, as it receives input from the median raphe nucleus, supramamillary nucleus, paraventricular nucleus, medial and lateral septal nuclei, and the diagonal band of Broca in both sexes^42–45^. The projection from the supramammillary nucleus was recently implicated in the encoding of novel social stimuli^46^ and controls the output of CA2 pyramidal cells by activating inhibitory interneurons in a feedforward manner^47^ (both studies conducted in male mice). Interestingly, while both male and female mice exhibit a preference for unfamiliar over familiar conspecifics, males spend more time investigating unfamiliar mice, which could be explained by a greater preference for social novelty^6^. Therefore, it is tempting to speculate that this preference might be encoded by stronger supramammillary input to CA2, resulting in increased spine head sizes in *stratum oriens* of males.

Sex hormones are also known to directly modulate synaptic function in the hippocampus^8,12^. Hippocampal neurons express several types of estrogen receptors (ERs). Classical ER signaling involves the activation of gene transcription via estrogen response elements in the DNA, but an additional, rapid form of signaling is mediated by plasma membrane-bound ERs^48^. The membrane ERs regulate synaptic plasticity by activating protein kinases such as Akt^48^, Src, and ERK1/2^49^, or, in the case of G-protein coupled ERs (GPERs), increasing the levels of postsynaptic density protein 95^50^. Differences in the membrane expression of ERs could account for the sex differences we observed in the spine size in CA2 *stratum radiatum*, since ERα was shown to be expressed at higher levels in CA1 *stratum radiatum* of female compared to male rats^49^. Furthermore, the expression levels of ERα and ERβ fluctuate with the estrous cycle in rats, but follow a different pattern in CA1 and CA3: ERα is expressed at the highest levels in CA1 during estrus and in CA3 during metestrus, whereas ERβ is highest in CA1 during metestrus and in CA3 during diestrus^51^. The authors attributed these differences to differences in the post-transcriptional regulation of the ERs or local estradiol synthesis^51^. In contrast, GPERs appear to be more highly expressed in *stratum oriens* of male compared to female rats, and in female rats in estrus compared to diestrus. The data for CA1 and CA3 and for *stratum radiatum* and *lacunosum-moleculare* were, however, pooled, so fine-grained differences between regions or layers may have been overlooked^52^.

While it was long believed that estrogens acting on the brain were of gonadal origin, it is now established that estradiol is also synthesized de novo from cholesterol in hippocampal neurons (reviewed in^53^). Neuron-derived estradiol plays an important role in LTP and hippocampal-dependent cognitive functions in both male and ovariectomized female mice, though the authors did not test social recognition memory^54^. However, in rats, the hippocampal levels of estradiol and progesterone correlate with the plasma levels^55^, likely due to the pulsative release of gonadotropin-releasing hormone from the hypothalamus which regulates both the estrous cycle and hippocampal estradiol synthesis^56^. Therefore, it is unlikely that the cycle-dependent differences in spine size and density in CA2 *stratum radiatum* are caused by differences in estradiol synthesis, since this would also be expected to increase the spine sizes in all dendritic layers. Although we can only speculate, a layer-specific difference in ER expression in CA2, in turn, would be consistent with our findings, and warrants a closer investigation in future studies.

Lastly, what might be the functional consequences of a higher spine density and/or a larger spine head? The vast majority of spines typically contain one excitatory synapse^22,57^, so spine density can function as a surrogate marker for excitatory synapse density. An increase in CA2 spine density, therefore, most likely impacts the degree of excitatory neurotransmission, and with it the processing of information in area CA2. Indeed, increased spine densities have been positively associated with enhanced performances in specific tasks in other brain regions (reviewed e.g. in^23^). It is therefore conceivable that the increased spine density found in *stratum radiatum* of diestrus females could be correlated with an increased performance in a CA2-specific memory task. A study by Sanchez–Andrade and Kendrick^58^ used the same habituation/dishabituation protocol for testing social memory in mice that was also used by Hitti and Siegelbaum^4^ to show that social recognition memory was abolished in CA2-silenced (but male) mice. The former analyzed female mice in the estrus, proestrus or diestrus stage of the estrous cycle, yet found them to perform similarly well in all stages. However, behavioral differences brought about by changes in spine density across the estrous cycle might be too subtle to be detected by such tests. The spine head volume is, via various positive correlations with postsynaptic density (PSD) area, number of presynaptic docked vesicles, and number of postsynaptic receptors, likely to be directly proportional to the average reliability and strength of its synapse^59^. While on average, large spines tend to be more resistant to structural potentiation^24^, serial section electron microscopy of dendrites in *stratum radiatum* of adult rats showed that a subset of large spines which contain smooth endoplasmic reticulum (SER) and/or polyribosomes exhibit the greatest PSD enlargement following the induction of long-term potentiation (LTP)^60^, a form of activity-dependent synaptic plasticity that is considered one of the main cellular correlates of long-term memory^61^. Thus, a greater proportion of large, presumably SER-containing spines in *stratum radiatum* of female mice in diestrus and in *stratum oriens* of male mice might indicate that many of these synapses have undergone LTP. However, the higher spine density we additionally observed in *stratum radiatum* during diestrus could represent a greater capacity for learning during this phase of the estrous cycle. It stands to reason that more challenging social recognition paradigms (e.g.^62^) might reveal subtle differences in learning ability during different phases of the estrous cycle.

CA2 is becoming an increasingly recognized hippocampal subregion known for its prominent role in social memory. We show here, for the first time, that apical and basal dendritic spines of identified CA2 neurons are influenced by sex hormones, and in addition are differentially affected. Our results provide a potential structural correlate to the sex-specific differences observed in certain CA2-dependent behavioral tasks, and add to the increasing body of evidence that both sex and stage of estrous are important biological variables that should be taken into consideration when interpreting morphological data.

## Methods

### Animals

Six male and 16 female young adult C57BL/6J mice aged between 10–14 weeks were used in this study. We have chosen this narrow age range in young adulthood to rule out potentially confounding age-related effects. The C57BL/6J mouse was chosen to ensure comparability with data from previous seminal CA2 studies. Animals were bought from Janvier labs, France, then maintained on a 12 h/12 h light–dark- cycle at the animal facility of the Goethe-University Hospital Frankfurt with water and food ad libitum until they were perfused. All mice were housed in same sex groups of 3–4 in individually ventilated type 2 Makrolon^®^ cages with a microbiological filter (Tecniplast, Buguggiate, Italy) and one water bottle, containing wood chip bedding and enriched with naturalistic nesting material as well as a mouse igloo (hard red plastic house). Experiments were conducted in accordance with the German Animal Welfare Act, and approved by the animal welfare officer of Goethe University Frankfurt, Faculty of Medicine and the Regierungspräsidium Darmstadt (FU/2039, FK/1126). Every effort was made to minimize distress and pain of the animals. Methods are reported in accordance with ARRIVE guidelines.

### Cycle staging via vaginal cytology

The phase of the estrous cycle was verified by vaginal smears as previously described^63^. Briefly, during the initial phase of the perfusion, which took place between 10 and 12 a.m., a wetted cotton swab was inserted into the vaginal opening and the smear was transferred onto a microscopic slide, stained with methylene blue solution and investigated microscopically (Axio Observer Z1, Carl Zeiss Microscopy). Diestrus and proestrus were determined using the following characterization: diestrus—epithelial cells and leucocytes observed; proestrus—large nucleated epithelial cells and no leucocytes observed (cf. Fig. 1a).

### Tissue preparation

Mice were killed with an overdose of pentobarbital (500 mg/kg KGW; Narcoren, intraperitoneal injection) or isoflurane, transcardially perfused with 0.1 M phosphate buffered saline (PBS) until the liver was completely bloodless, and then fixed with 4% paraformaldehyde (PFA) in 0.1 M PBS for 5 min. Brains were taken out immediately after perfusion and post-fixed for 18 h in the same fixative at 4 °C. Brains were washed thrice in ice-cold 0.1 M PBS and sectioned (250 µm coronal slices) on a vibratome (Leica VT 1000 S) using a custom-built object holder to tilt the brain in an approx. 30° angle to account for the branching of CA2 neurons along the transverse axis of the hippocampus. Brains were only prepared on demand and cut within 2 days after perfusion. Sections were stored in 0.1 M PBS at 4 °C and used for fillings during the next 4 days.

### Intracellular post-fixation filling

Intracellular injections of dorsal CA2 neurons in fixed slices were performed as previously described^64,65^ with modifications. Sharp quartz-glass microelectrodes (Sutter Instruments, QF100-70-10, with filament) were pulled using a P-2000 laser puller (Sutter Instruments). Microelectrodes were loaded with 0.5 mM Alexa Fluor 568 Hydrazide (Invitrogen) in HPLC-grade water and then back-filled with 0.1 M LiCl in HPLC-grade water. Sections were incubated for 10 min in the nuclear stain Hoechst diluted 1:5000 in 0.1 M PBS to stain nuclei and visualize hippocampal morphology, then washed twice for 5 min in 0.1 M PBS. Sections were then placed in a custom-made chamber filled with cold PBS and mounted on the fixed stage of an Olympus BX51WI upright microscope equipped with a 10× water immersion objective (LMPlanFLN10× , NA 0.25, WD 21 mm), placed on an x–y translation table (Science Products). The hippocampal CA2 region was visually identified using the Hoechst stain. Using a motorized 3D micromanipulator, the electrode was lowered into the hippocampal CA2 region under visual control while applying a negative voltage pulse (− 1 V, 1 Hz) to the electrode via a silver wire in line with a 500 MOhm resistor. When piercing of a cell body was observed, the cell was filled by application of a negative 1 Hz current pulse to the electrode. Filling was for 10 min or until no further filling was observed (Fig. 1b). Afterwards, slices were fixed for one to 2 days in 4% PFA at 4 °C and washed three times in cold 0.1 M PBS.

### Post-hoc immunohistochemistry

Filled and fixed injected sections (250 µm) were embedded in 5% agar and resliced to 40 µm-thin sections on a vibratome (Leica VT 1000 S) to facilitate staining. Free-floating sections were washed three times in 50 mM Tris-buffered saline (TBS) containing 0.1% Triton X-100, incubated in a blocking buffer (0.5% Triton X-100, 5% bovine serum albumin (BSA) in 50 mM TBS) for 30 min at room temperature (RT) and subsequently incubated with rabbit anti-PCP4 antibody (1:1000; Sigma–Aldrich Cat# HPA005792, RRID: AB_1855086) diluted in 0.1% Triton X-100 + 1% BSA in 50 mM TBS for 3 days at RT. After four washing steps, sections were incubated with goat anti-rabbit Alexa Fluor 647 secondary antibody (1:1000, Thermo Fisher Scientific Cat# A-21245, RRID: AB_2535813) for 4 h at RT, washed four times in 50 mM TBS, and mounted on glass slides with Dako fluorescence mounting medium. PCP4 staining was consistent with published data^1^.

### Image acquisition

An Alexa Fluor 568-filled neuron was considered a CA2 cell if the cell body was PCP4-positive (Fig. 1d). Dendritic segments (3rd to 6th order for SR; ≥ 3rd order for SO) associated with an identified CA2 neuron were imaged using an Olympus FV1000 microscope and a 60× oil-immersion objective (UPlanSApo, NA 1.35, Olympus) using FV10-ASW software with 5× scan zoom at a resolution of 1024 × 1024 pixels and 0.25 µm z-distance. Imaging parameters were set for each segment individually to capture it as brightly as possible without oversaturating the spines.

### Image processing and data analysis

To be considered for analysis, segments had to fulfill the following criteria: (1) belong to a PCP4-stained neuron; (2) ≥ 20 µM long; (3) evenly filled with dye, signal-to-noise-ratio sufficient for deconvolution. Confocal image stacks were deconvolved using Huygens professional version 17.10 (Scientific Volume Imaging, The Netherlands). One segment per labeled cell with the best post-deconvolution quality was chosen for subsequent analysis. FIJI^66^ was then used for image processing and raw data analysis. Analysis was done with the investigator(s) blind to sex or cycle stage. Spine density of SO and SR was partially analyzed by two independent investigators (SJ+MF), yielding comparable average results. Data obtained by SJ are presented in this paper.

Spine analysis was performed following the criteria of Holtmaat^67^ with minor modifications: dendritic spines of all shapes were assessed manually in 3D by scrolling through the z-stacks. Only protrusions emanating laterally in the x–y directions and exceeding the dendritic shaft for at least 0.2 µm (5 pixels) were included in the analysis. The length of each segment was measured in 3D using the ImageJ plugin SimpleNeuriteTracer^68^. Spine density was expressed as spines per µm dendrite. For spine head size measurements, the brightness of each image was individually adjusted to saturate 0.1% of the pixels in the shaft of the dendritic segment to be analyzed. Maximum cross-sectional head area of a spine was then measured by manually circling the heads using the same predefined gray-value as a cut-off for all segments. Only spines with a discernible head were included in this analysis.

### Statistical analysis

Data were analyzed and visualized using GraphPad Prism 6. Averaged data are presented as whisker-box-plot, with upper/lower whisker representing maximum/minimum values, box representing median with upper and lower quartile, cross representing mean. The Kruskal–Wallis test was used to compare averaged data of the three groups (male/female diestrus/female proestrus). In case of significance (*P* < 0.05), Dunn’s post-hoc comparison test was employed. Corresponding statistical values in the text are expressed as mean ± standard deviation (s.d.). For frequency distribution data, the Kolmogorov–Smirnov test was used for pairwise comparisons of the groups. *P < 0.05, **P < 0.01, ***P < 0.001, ****P < 0.0001. *Stratum oriens*—male: 1209 total spines, 883 spines with head, on 14 segments from six animals; female diestrus: 1697 total spines, 1297 spines with head, on 18 segments from seven animals; female proestrus: 1638 total spines, 1182 spines with head, on 17 segments from eight animals. *Stratum radiatum*—male: 1597 total spines, 1156 spines with head, on 15 segments from five animals; female diestrus: 971 total spines, 667 spines with head, on eight segments from four animals; female proestrus: 1965 total spines, 1534 spines with head, on 15 segments from five animals.

## Data Availability

Data is available from the corresponding author upon reasonable request.
